# Supplementary material for: Iterative improvement in the automatic modular design of robot swarms
Source: PeerJ Comput Sci. 2020 Dec 7;6:e322. doi: 10.7717/peerj-cs.322 (PMC7924708; doi:10.7717/peerj-cs.322)
Supplement: Supplemental Information 3 [file peerj-cs-06-322-s003.zip › argos3/doc/api/standalone/a00536_source.html]

ARGoS: plugins/robots/generic/simulator/proximity\_default\_sensor.cpp Source File


- Main Page
- Related Pages
- Namespaces
- Classes
- Files

- File List
- File Members

# plugins/robots/generic/simulator/proximity\_default\_sensor.cpp

Go to the documentation of this file.

```
00001 
00007 #include <argos3/core/simulator/simulator.h>
00008 #include <argos3/core/simulator/entity/embodied_entity.h>
00009 #include <argos3/core/simulator/entity/composable_entity.h>
00010 #include <argos3/plugins/simulator/entities/proximity_sensor_equipped_entity.h>
00011 
00012 #include "proximity_default_sensor.h"
00013 
00014 namespace argos {
00015 
00016    /****************************************/
00017    /****************************************/
00018 
00019    static CRange<Real> UNIT(0.0f, 1.0f);
00020 
00021    /****************************************/
00022    /****************************************/
00023 
00024    CProximityDefaultSensor::CProximityDefaultSensor() :
00025       m_pcEmbodiedEntity(NULL),
00026       m_bShowRays(false),
00027       m_pcRNG(NULL),
00028       m_bAddNoise(false),
00029       m_cSpace(CSimulator::GetInstance().GetSpace()) {}
00030 
00031    /****************************************/
00032    /****************************************/
00033 
00034    void CProximityDefaultSensor::SetRobot(CComposableEntity& c_entity) {
00035       try {
00036          m_pcEmbodiedEntity = &(c_entity.GetComponent<CEmbodiedEntity>("body"));
00037          m_pcControllableEntity = &(c_entity.GetComponent<CControllableEntity>("controller"));
00038          m_pcProximityEntity = &(c_entity.GetComponent<CProximitySensorEquippedEntity>("proximity_sensors"));
00039          m_pcProximityEntity->Enable();
00040       }
00041       catch(CARGoSException& ex) {
00042          THROW_ARGOSEXCEPTION_NESTED("Can't set robot for the proximity default sensor", ex);
00043       }
00044    }
00045 
00046    /****************************************/
00047    /****************************************/
00048 
00049    void CProximityDefaultSensor::Init(TConfigurationNode& t_tree) {
00050       try {
00051          CCI_ProximitySensor::Init(t_tree);
00052          /* Show rays? */
00053          GetNodeAttributeOrDefault(t_tree, "show_rays", m_bShowRays, m_bShowRays);
00054          /* Parse noise level */
00055          Real fNoiseLevel = 0.0f;
00056          GetNodeAttributeOrDefault(t_tree, "noise_level", fNoiseLevel, fNoiseLevel);
00057          if(fNoiseLevel < 0.0f) {
00058             THROW_ARGOSEXCEPTION("Can't specify a negative value for the noise level of the proximity sensor");
00059          }
00060          else if(fNoiseLevel > 0.0f) {
00061             m_bAddNoise = true;
00062             m_cNoiseRange.Set(-fNoiseLevel, fNoiseLevel);
00063             m_pcRNG = CRandom::CreateRNG("argos");
00064          }
00065          m_tReadings.resize(m_pcProximityEntity->GetNumSensors());
00066       }
00067       catch(CARGoSException& ex) {
00068          THROW_ARGOSEXCEPTION_NESTED("Initialization error in default proximity sensor", ex);
00069       }
00070    }
00071 
00072    /****************************************/
00073    /****************************************/
00074    
00075    void CProximityDefaultSensor::Update() {
00076       /* Ray used for scanning the environment for obstacles */
00077       CRay3 cScanningRay;
00078       CVector3 cRayStart, cRayEnd;
00079       /* Buffers to contain data about the intersection */
00080       SEmbodiedEntityIntersectionItem sIntersection;
00081       /* Go through the sensors */
00082       for(UInt32 i = 0; i < m_tReadings.size(); ++i) {
00083          /* Compute ray for sensor i */
00084          cRayStart = m_pcProximityEntity->GetSensor(i).Offset;
00085          cRayStart.Rotate(m_pcProximityEntity->GetSensor(i).Anchor.Orientation);
00086          cRayStart += m_pcProximityEntity->GetSensor(i).Anchor.Position;
00087          cRayEnd = m_pcProximityEntity->GetSensor(i).Offset;
00088          cRayEnd += m_pcProximityEntity->GetSensor(i).Direction;
00089          cRayEnd.Rotate(m_pcProximityEntity->GetSensor(i).Anchor.Orientation);
00090          cRayEnd += m_pcProximityEntity->GetSensor(i).Anchor.Position;
00091          cScanningRay.Set(cRayStart,cRayEnd);
00092          /* Compute reading */
00093          /* Get the closest intersection */
00094          if(GetClosestEmbodiedEntityIntersectedByRay(sIntersection,
00095                                                      cScanningRay,
00096                                                      *m_pcEmbodiedEntity)) {
00097             /* There is an intersection */
00098             if(m_bShowRays) {
00099                m_pcControllableEntity->AddIntersectionPoint(cScanningRay,
00100                                                             sIntersection.TOnRay);
00101                m_pcControllableEntity->AddCheckedRay(true, cScanningRay);
00102             }
00103             m_tReadings[i] = CalculateReading(cScanningRay.GetDistance(sIntersection.TOnRay));
00104          }
00105          else {
00106             /* No intersection */
00107             m_tReadings[i] = 0.0f;
00108             if(m_bShowRays) {
00109                m_pcControllableEntity->AddCheckedRay(false, cScanningRay);
00110             }
00111          }
00112          /* Apply noise to the sensor */
00113          if(m_bAddNoise) {
00114             m_tReadings[i] += m_pcRNG->Uniform(m_cNoiseRange);
00115          }
00116          /* Trunc the reading between 0 and 1 */
00117          UNIT.TruncValue(m_tReadings[i]);
00118       }
00119    }
00120 
00121    /****************************************/
00122    /****************************************/
00123 
00124    void CProximityDefaultSensor::Reset() {
00125       for(UInt32 i = 0; i < GetReadings().size(); ++i) {
00126          m_tReadings[i] = 0.0f;
00127       }
00128    }
00129 
00130    /****************************************/
00131    /****************************************/
00132 
00133    Real CProximityDefaultSensor::CalculateReading(Real f_distance) {
00134       return Exp(-f_distance);
00135    }
00136 
00137    /****************************************/
00138    /****************************************/
00139 
00140    REGISTER_SENSOR(CProximityDefaultSensor,
00141                    "proximity", "default",
00142                    "Carlo Pinciroli [ilpincy@gmail.com]",
00143                    "1.0",
00144                    "A generic proximity sensor.",
00145                    "This sensor accesses a set of proximity sensors. The sensors all return a value\n"
00146                    "between 0 and 1, where 0 means nothing within range and 1 means an external\n"
00147                    "object is touching the sensor. Values between 0 and 1 depend on the distance of\n"
00148                    "the occluding object, and are calculated as value=exp(-distance). In\n"
00149                    "controllers, you must include the ci_proximity_sensor.h header.\n\n"
00150                    "REQUIRED XML CONFIGURATION\n\n"
00151                    "  <controllers>\n"
00152                    "    ...\n"
00153                    "    <my_controller ...>\n"
00154                    "      ...\n"
00155                    "      <sensors>\n"
00156                    "        ...\n"
00157                    "        <proximity implementation=\"default\" />\n"
00158                    "        ...\n"
00159                    "      </sensors>\n"
00160                    "      ...\n"
00161                    "    </my_controller>\n"
00162                    "    ...\n"
00163                    "  </controllers>\n\n"
00164                    "OPTIONAL XML CONFIGURATION\n\n"
00165                    "It is possible to draw the rays shot by the proximity sensor in the OpenGL\n"
00166                    "visualization. This can be useful for sensor debugging but also to understand\n"
00167                    "what's wrong in your controller. In OpenGL, the rays are drawn in cyan when\n"
00168                    "they are not obstructed and in purple when they are. In case a ray is\n"
00169                    "obstructed, a black dot is drawn where the intersection occurred.\n"
00170                    "To turn this functionality on, add the attribute \"show_rays\" as in this\n"
00171                    "example:\n\n"
00172                    "  <controllers>\n"
00173                    "    ...\n"
00174                    "    <my_controller ...>\n"
00175                    "      ...\n"
00176                    "      <sensors>\n"
00177                    "        ...\n"
00178                    "        <proximity implementation=\"default\"\n"
00179                    "                   show_rays=\"true\" />\n"
00180                    "        ...\n"
00181                    "      </sensors>\n"
00182                    "      ...\n"
00183                    "    </my_controller>\n"
00184                    "    ...\n"
00185                    "  </controllers>\n\n"
00186                    "It is possible to add uniform noise to the sensors, thus matching the\n"
00187                    "characteristics of a real robot better. This can be done with the attribute\n"
00188                    "\"noise_level\", whose allowed range is in [-1,1] and is added to the calculated\n"
00189                    "reading. The final sensor reading is always normalized in the [0-1] range.\n\n"
00190                    "  <controllers>\n"
00191                    "    ...\n"
00192                    "    <my_controller ...>\n"
00193                    "      ...\n"
00194                    "      <sensors>\n"
00195                    "        ...\n"
00196                    "        <proximity implementation=\"default\"\n"
00197                    "                   noise_level=\"0.1\" />\n"
00198                    "        ...\n"
00199                    "      </sensors>\n"
00200                    "      ...\n"
00201                    "    </my_controller>\n"
00202                    "    ...\n"
00203                    "  </controllers>\n\n",
00204                    "Usable"
00205                   );
00206 
00207 }
```

---

Generated on 10 Jul 2018 for ARGoS by 
 1.6.1
